# How to design and deliver a local teaching program

**DOI:** 10.1097/IJ9.0000000000000023

**Published:** 2017-06-08

**Authors:** Christopher Limb, Katharine Whitehurst, Buket Gundogan, Kiron Koshy, Riaz Agha

**Affiliations:** a St Helier's Hospital, Surrey; b Royal Devon and Exeter Hospital; c University College London Medical School; d Brighton and Sussex University Hospitals; e Guy's and St Thomas' NHS Foundation Trust, London, UK

**Keywords:** How to, Teaching, Teaching program, Teach

## Abstract

Teaching is an invaluable aspect of any medical or surgical career. Many trainees will find themselves delivering teaching at several stages in their career and in this “How to” article we explain how to design, set up, and deliver a successful teaching program, as well as how to evidence this in your portfolio.

Involvement in teaching is an invaluable aspect of any medical or surgical career. Not only is it a rewarding experience and good for personal development, but it also contributes to interview scoring throughout surgery and medicine, and subsequently your future career. For example in the teaching component of a Core Surgical portfolio: 3 points are achieved for having “Designed and led local teaching, with positive feedback and evidence of refection,” with the full 5 points for having “Designed, organized, and delivered more than 1 teaching program and extensive formal training in teaching methodology”^1^.

In this article, we will not review the different approaches to teaching, but explain how to go about leading the design and delivery of a successful teaching program, and how to evidence this in your portfolio. We will describe the basic framework in setting up a local teaching program, which can be extrapolated to the size and audience you feel you can manage.

## Planning

The most important part in delivering a successful teaching program is planning, from an early stage, all aspects of the program: including target audience, content, timing, and team to be involved.

### Target audience

It is often most appropriate to offer teaching for those at the same or early stages of their study/work. This allows you to provide sessions that will be useful, and also means people will have the desire to attend. For example, a first year student could feel they would benefit from teaching sessions provided by a final year, but very rarely the other way around. Look for gaps in the current educational curriculum: if there is limited time for practical OSCE skills this could be a useful program to develop.

### Content

As above, consider what styles of teaching are lacking, but also consider what you and your team would be most able to teach. For example, I believe my own clinical skills are better than my knowledge, so in my last teaching program I delivered bedside teaching as opposed to lectures or seminars. Consider reviewing the various approaches to teaching and choosing what you think you would be able to lead best. Your content should cover a topic area, for example, if providing sessions on OSCE skills try to spend enough time on each system without leaving gaps.

## Timing

A good teaching program should cover most important areas; so make sure you have enough time to complete it before you move placement or job. As a rough guide: aim for between 6 and 10 sessions. Give yourself 2–4 weeks to plan, promote, and prepare for the program to start. But again this depends on your own commitment. One weekly session is often most easily managed, both from the provision side and also to get regular attenders.

## Team

To lead the program effectively you need an appropriate team to help plan, promote, and deliver the teaching sessions. Ask your peers if they would like to be involved early and meet to plan the teaching early. This is a great opportunity for others to be involved in teaching, people often make valuable contributions you have missed and make the work load manageable.

Finally, it is very useful to have senior support, preferably your tutor or educational supervisor. Not only may they be able to point you toward useful sessions to run, but at the end they can give evidence for your involvement.

### Working as a team

As the leader it is important for you to fairly and appropriately divide the preparation for each session between the team. Each session may only require 1 person to deliver or require the support of the whole team: make a plan for who is going to lead the teaching on each day and who else can and should be there. Don’t take all of the work yourself, it will be too much to manage and take potential learning opportunities from other members of your team.

Ask the members what they are best at and try to accommodate this when allocating work. Make sure that if any member is struggling they let you know early so that you can help or appoint another team member to assist.

## Promotion

Now that you have designed, with the help of your team, a good teaching program you need to make sure the target audience is aware! There are numerous ways this can be done and depend on your specific location but here are a few ideas:

- Speak to the mailing list coordinator, for example, at the undergraduate or postgraduate department, and ask for an email to be sent to the target audience.
- Ask to speak at the end of a lecture or seminar.
- Put up posters where they will be seen, explaining what, where, and when you will be offering this teaching.
- Speak to representatives of the target audience.
- Word of mouth.

Try to get feedback from members of the target audience as to whether they have heard of your program, and if not what else you could do to promote it.

## Feedback

No matter how well your teaching sessions goes it is important to obtain feedback: this allows you to adapt to the audience between sessions and improve the teaching you provide. It is also very important for your portfolio, remember that for 3 points it must be “with positive feedback,” and don’t worry: with a good teaching program the feedback will invariably be positive over all.

The undergraduate and postgraduate offices are generally able to provide feedback tools, however, and tool with sessional information (such as location, date, and tutors) and feedback (considering both what went well and what could be improved) is appropriate.

## Review progress

Take the time to speak to each member of the team, particularly before they lead a session, to make sure they have not encountered any issues and for feedback. This means that you can spot any problems early on to avoid them and react to make any changes that could improve your session. Speak to your supervisor during the program to discuss its progression and for help on any other issues you encounter.

## Reflection

During and at the end of your teaching program it is important to take the time to reflect. This is discussed in more detail in our article on “reflective practice”; but to summarize it gives you the opportunity to gain insight into why things went well, or not so well, and the opportunity to develop yourself so you will become a better teacher and lead in your future roles. Utilize formal tools, such as the LEADER framework on the ePortfolio, and involve your supervisor to provide insight you may not have appreciated and consolidate the experience. Again, formal recording of these meetings is useful evidence in your portfolio: for 3 marks you must provide “evidence of refection.”
